# R-Wave Peak Time and Impaired Coronary Collateral Circulation in Chronic Total Occlusion

**DOI:** 10.3390/jcm15020450

**Published:** 2026-01-07

**Authors:** Nadir Emlek, Hüseyin Durak, Mustafa Çetin, Ali Gökhan Özyıldız, Elif Ergül, Ahmet Seyda Yılmaz, Hakan Duman

**Affiliations:** Department of Cardiology, Faculty of Medicine, Recep Tayyip Erdoğan University, İslampaşa Mah., Şehitler Cd. No:74, 53020 Rize, Turkey

**Keywords:** electrocardiography, R-wave peak time, chronic total occlusion, coronary collateral circulation

## Abstract

**Background/Objectives:** Chronic total occlusion (CTO) is one of the most complex forms of coronary artery disease, and myocardial perfusion in patients with CTO largely depends on the adequacy of coronary collateral circulation (CCC). Identifying simple and non-invasive electrocardiographic markers associated with impaired collateralization remains clinically important. The R-wave peak time (RWPT), a surface electrocardiography (ECG) marker representing the time to peak R-wave deflection and an electrocardiographic surrogate of early intraventricular conduction, may provide insight into ischemia-related ventricular activation delay. The aim of this study was to evaluate whether RWPT is associated with poor CCC in patients with CTO. **Methods:** This cross-sectional observational study included 92 consecutive patients with CTO and complete clinical, angiographic, and 12-lead ECG data. Patients were categorized according to CCC adequacy into good (*n* = 52) and poor (*n* = 40) CCC groups. Demographic, laboratory, angiographic, and ECG parameters were compared. Variables showing significant differences were subjected to univariate analysis, followed by multivariate logistic regression using a backward stepwise selection method. Statistical significance was set at *p* < 0.05. **Results:** Patients with poor CCC were significantly older and exhibited longer QRS duration and prolonged RWPT, whereas triglyceride levels were significantly lower. In multivariate analysis, both age (OR: 1.058; 95% CI: 1.005–1.114; *p* = 0.033) and RWPT (OR: 1.069; 95% CI: 1.013–1.128; *p* = 0.015) were significantly associated with poor CCC. **Conclusions:** RWPT may provide adjunctive, non-invasive information regarding collateral adequacy rather than serving as a definitive predictive marker. As an easily obtainable ECG parameter, RWPT may offer incremental diagnostic information when interpreted alongside clinical and angiographic findings in patients with CTO.

## 1. Introduction

Chronic total occlusion (CTO) is defined as a completely obstructive coronary artery lesion with an estimated duration of at least three months and Thrombolysis in Myocardial Infarction (TIMI) grade 0–1 antegrade flow in the distal vessel [1]. Percutaneous coronary intervention (PCI) for CTO is one of the most complex procedures in interventional cardiology because it is technically demanding and is associated with a higher risk of procedural complications. In patients with clinically significant coronary artery disease, CTO is reported to be present in approximately 18% of diagnostic coronary angiograms [2]. Coronary collateral circulation (CCC) refers to natural bypass channels that develop within the same coronary artery or between different coronary arteries to maintain perfusion of the distal myocardium in the setting of critical stenosis or total occlusion [3].

The preservation of myocardial perfusion in the presence of CTO largely depends on the adequacy of the CCC. Good CCC has been associated with limitations of ischemia, reduction in infarct size, prevention of left ventricular aneurysm, improved post-infarction left ventricular function, and decreased long-term cardiovascular mortality. In contrast, poor CCC increases the severity of ischemia, accelerates the deterioration of left ventricular function, and leads to unfavorable outcomes.

Although factors influencing CCC have been investigated for many years, risk factors such as hypercholesterolemia, advanced age, hypertension, diabetes, obesity, smoking, and genetic predisposition have been shown to impair angiogenic responses and negatively affect CCC development [4]. However, a practical, low-cost, and non-invasive marker capable of rapidly predicting the status of CCC is lacking in clinical practice.

Electrocardiography (ECG) is a widely used and easily accessible diagnostic method that provides valuable information about myocardial conduction properties. In recent years, several ECG parameters, particularly QRS duration, fragmented QRS (fQRS), pathologic Q waves, and R-wave peak time (RWPT), have been shown to be associated with myocardial structural integrity and ischemic burden [5,6,7,8].

RWPT is a measure that reflects the ventricular activation time and represents the conduction velocity of the electrical impulse from the endocardium to the epicardium [9]. Previous studies have reported several clinical implications of RWPT, noting that its prolongation is particularly associated with left ventricular hypertrophy, myocardial ischemia, and intraventricular conduction abnormalities [9,10,11].

The relationship between various ECG parameters, such as fQRS, QRS duration, QT dispersion, and corrected QT (QTc) dispersion, and poor CCC has been investigated in only a limited number of studies to date [12,13,14,15]. However, in most of these studies, the independent effect of RWPT on CCC was not clearly demonstrated. Because RWPT reflects myocardial ischemia and electrical activation delay, it may serve as a potential non-invasive marker of CCC adequacy in patients with CTO.

Therefore, evaluating the relationship between RWPT measured on a standard 12-lead surface ECG and the presence of poor CCC in patients with CTO represents a clinically relevant and timely research question. If a simple ECG parameter can provide information about CCC adequacy without the need for invasive assessment, it may offer a meaningful advantage in the management of patients.

In patients with CTO, CCC adequacy is a key determinant of preserved myocardial perfusion and clinical outcomes. However, evidence regarding reliable non-invasive predictors of CCC remains limited. RWPT, measured on a surface ECG, is an easily applicable and low-cost parameter that reflects the early ventricular depolarization delay. This study aimed to evaluate the association between RWPT and poor coronary collateral circulation in patients with CTO and to explore its potential role as an adjunctive electrocardiographic parameter reflecting collateral adequacy.

## 2. Materials and Methods

### 2.1. Study Design and Population

This was a single-center, observational, cross-sectional study. A total of 92 consecutive patients from Recep Tayyip Erdoğan University Hospital who underwent coronary angiography (CAG) for stable angina pectoris and were diagnosed with CTO were included. The medical history, physical examination findings, 12-lead ECG results, transthoracic echocardiography data, CAG findings, concomitant systemic diseases, and current medications were recorded for all patients. Body mass index (BMI) was calculated by dividing body weight in kilograms by the square of the height in meters.

Written informed consent was obtained from all participants prior to enrollment. The study protocol was approved by the Ethics Committee of Recep Tayyip Erdoğan University (Non-Interventional Clinical Research Ethics Committee, Approval Date: 2 June 2024, Approval No: 2024-217).

### 2.2. Inclusion and Exclusion Criteria

Patients with a confirmed diagnosis of CTO, complete ECG evaluation, and comprehensive laboratory data were included in the study. The exclusion criteria were as follows: acute coronary syndrome, malignancy, atrial fibrillation, connective tissue diseases, advanced hepatic or renal failure, pericarditis, presence of a pacemaker, history of coronary artery bypass grafting (CABG), surgical valve replacement, cardiomyopathy, and congenital heart disease.

### 2.3. Laboratory Measurements

Peripheral venous blood samples were obtained from all patients after a 12-h overnight fast for complete blood count and biochemical analyses. Lipid profiles, fasting glucose, and creatinine levels were measured using chemiluminescence. Complete blood count samples were collected in EDTA-containing tubes and analyzed using an automated hematology analyzer (Beckman Coulter, Brea, CA, USA).

### 2.4. Transthoracic Echocardiography

Transthoracic echocardiography was performed by an experienced cardiologist using a Philips Epiq 7 system (Philips, Andover, MA, USA) equipped with a 1–5 MHz X5-1 transducer. Parasternal long- and short-axis views and apical two- and four-chamber images were obtained using simultaneous ECG monitoring. The left ventricular ejection fraction was calculated using the modified Simpson method, in accordance with the recommendations of the American Society of Echocardiography.

### 2.5. Coronary Imaging

CAG was performed using the standard Judkins technique. The left anterior descending (LAD) and circumflex (CX) arteries were imaged in at least four projections, and the right coronary artery (RCA) was imaged in at least two projections. Lesions with 100% luminal occlusion in the relevant coronary artery and symptoms persisting for at least three months were considered CTO. The CCC of the CTO vessel was assessed according to the Rentrop classification [16]. Rentrop grades 0–1 were defined as poor CCC, and grades 2–3 indicated good CCC. Although the Rentrop classification is widely used in clinical and research settings, it primarily reflects angiographic visualization of collateral vessels rather than functional collateral flow.

### 2.6. Electrocardiographic Evaluation

A standard 12-lead ECG (150 Hz filter, 25 mm/s, 10 mm/mV; Schiller Cardiovit AT-10, Baar, Switzerland) was obtained for all patients. RWPT was measured in milliseconds as the interval from the onset of the R wave to its peak on the QRS complex [17]. Additional ECG parameters included interatrial block, QRS duration, fQRS, presence of pathologic Q waves, P-wave dispersion, QT interval, QTc interval, Tpeak to Tend (Tp-e) interval, and P-wave terminal force in V1 (PTF-V1). The comprehensive methodology for assessing each ECG parameter is detailed in the Supplementary File S1. All measurements were independently evaluated in a blinded manner by two cardiologists, and the average values were recorded.

### 2.7. Statistical Analysis

Continuous variables were assessed using visual methods (histograms and probability plots) and analytical tests (Kolmogorov–Smirnov and Shapiro–Wilk tests) to evaluate normality. Categorical variables are presented as percentages, and continuous variables as mean ± standard deviation. To compare categorical variables between groups, the chi-square test or Fisher’s exact test was used (the latter was applied when expected cell counts were insufficient for the chi-square test). Normally distributed continuous parameters were analyzed using one-way analysis of variance (ANOVA). After the two groups were compared (Table 1), variables that demonstrated statistically significant differences were first examined using univariate logistic regression analysis. Subsequently, the variables that remained significant were entered into a model for multivariate logistic regression analysis (Table 2). Given the relatively limited number of events (*n* = 40 patients with poor CCC), the multivariable logistic regression model was intentionally restricted to a small number of clinically relevant variables that showed significance in univariate analysis in order to reduce the risk of overfitting. Given the potential influence of heart rate on RWPT, heart rate–corrected R-wave peak time was calculated using the Fridericia formula and used in the primary analyses. A *p*-value < 0.05 was considered statistically significant.

Sensitivity analyses were performed to assess the robustness of the association between R-wave peak time and poor coronary collateral circulation. These analyses included (i) modeling RWPT as a continuous variable using different heart rate correction formulas (Fridericia and Bazett), and (ii) evaluating RWPT without correction. Consistency of effect estimates across models was used to support the stability of the findings.

## 3. Results

A total of 92 patients with CTO were included in this analysis. Based on angiographic evaluation, 52 patients had good CCC, whereas 40 had poor CCC. The baseline demographic, clinical, laboratory, and ECG characteristics of the two groups are presented in Table 1. Baseline echocardiographic assessment demonstrated that patients with poor CCC had significantly lower left ventricular ejection fraction and larger LV end-diastolic diameter compared with those with good CCC. LV end-systolic diameter showed a borderline difference, whereas interventricular septal and posterior wall thicknesses were similar between groups (Table 1).

**Figure 1:**
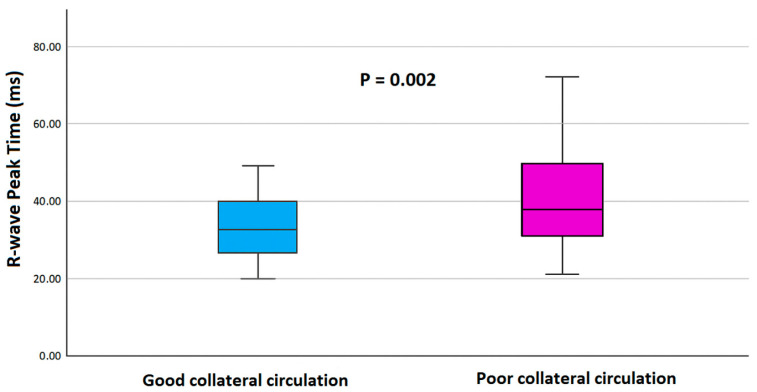
Boxplot comparison of R-wave peak time (RWPT) between patients with good and poor coronary collateral circulation (CCC). RWPT was significantly higher in the poor CCC group (*p* = 0.002).

Patients in the poor CCC group were significantly older (*p* = 0.043) and exhibited longer QRS duration (*p* = 0.021) and prolonged RWPT values (*p* = 0.002). In contrast, triglyceride levels were significantly lower in the poor CCC group (*p* = 0.003). Although not statistically significant, the prevalence of pathologic Q waves (*p* = 0.084) and the number of CTO lesions (*p* = 0.082) tended to be higher in patients with poor CCC. As shown in Figure 1, RWPT values were significantly higher in the poor CCC group (*p* = 0.002).

**Figure 2:**
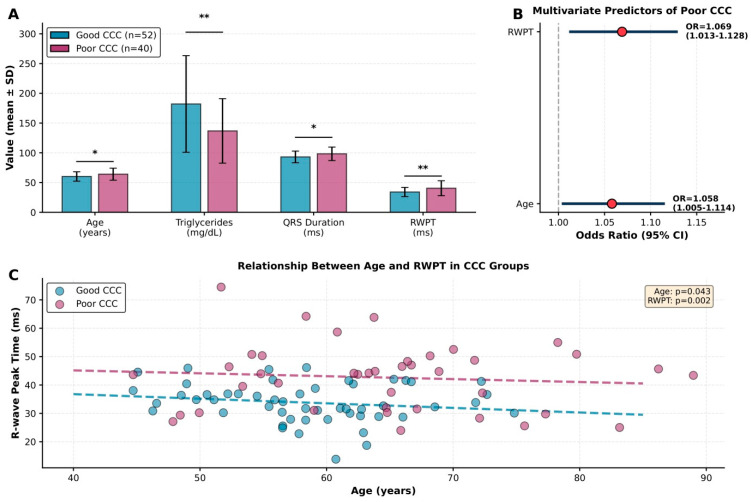
Panel (**A**): Comparison of statistically significant variables, bar charts showing mean ± SD for the four statistically significant continuous variables, statistical significance indicated with asterisks (* *p* < 0.05, ** *p* < 0.01); Panel (**B**): Forest plot of multivariate predictors, displays the two independent predictors from multivariate logistic regression. Red circles indicate point estimates of the odds ratios, and horizontal lines represent the 95% confidence intervals. The vertical dotted line indicates the line of no effect (OR = 1.0), whereas the faint vertical lines are gridlines used for visual reference only; Panel (**C**): Scatter plot with trend lines, shows the relationship between age and RWPT in both groups. Dashed lines represent group-specific linear regression trends.

**Figure 3:**
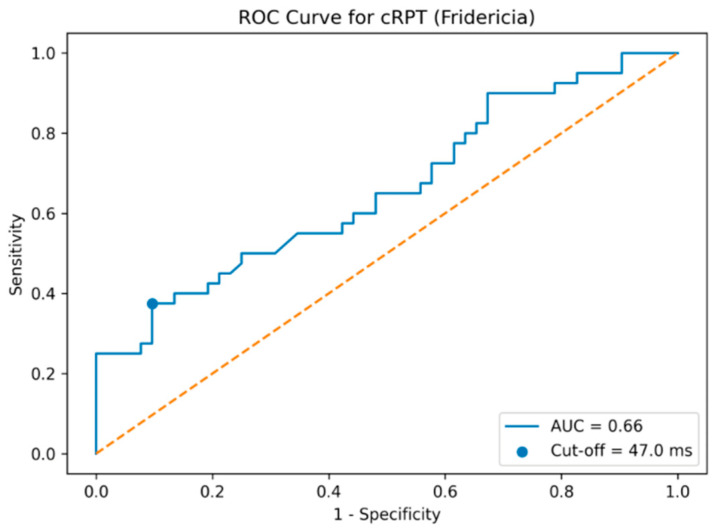
Receiver operating characteristic (ROC) curve of Fridericia-corrected R-wave peak time for identifying poor collateral flow. The dotted diagonal line represents the line of no discrimination (AUC = 0.5).

In the univariate logistic regression analysis, age (OR 1.047, 95% CI 1.001–1.098; *p* = 0.037), triglyceride levels (OR 0.989, 95% CI 0.982–0.997; *p* = 0.007), QRS duration (OR 1.071, 95% CI 1.022–1.123; *p* = 0.004), and RWPT (OR 1.049, 95% CI 1.006–1.093; *p* = 0.025) were significantly associated with a poor CCC (Figure 2). In the multivariate (backward) logistic regression model, only age (OR 1.058, 95% CI 1.005–1.114; *p* = 0.033) and RWPT (OR 1.069, 95% CI 1.013–1.128; *p* = 0.015) remained independently associated with poor CCC (Figure 2). Receiver operating characteristic (ROC) curve analysis was performed to assess the discriminative ability of Fridericia-corrected R-wave peak time (cRPT) for identifying poor coronary collateral circulation. cRPT demonstrated a moderate discriminative ability (Figure 3).

In addition, a parsimonious multivariable logistic regression model was constructed to explore variables independently associated with poor coronary collateral circulation (Table 3).

Receiver operating characteristic (ROC) curve analysis was performed to assess the discriminative ability of Fridericia-corrected R-wave peak time (cRPT) for identifying poor coronary collateral circulation. As shown in Figure 3, cRPT demonstrated a moderate discriminative ability, with an area under the curve (AUC) of 0.66.

In bootstrap analysis with 2000 resamples, the area under the ROC curve for Fridericia-corrected R-wave peak time was 0.66 with a 95% confidence interval ranging from 0.54 to 0.77. An exploratory cut-off value of ≥47.0 ms was identified using the Youden index.

In the final parsimonious multivariable model including age, triglyceride level, and Fridericia-corrected R-wave peak time, age remained independently associated with poor coronary collateral circulation (Table 3). Sensitivity analyses using alternative RWPT modeling approaches, including uncorrected RWPT and heart rate–corrected RWPT using Fridericia and Bazett formulas, yielded comparable effect sizes and directions, with RWPT remaining positively associated with poor CCC across models. Given the limited number of events, the multivariable model was deliberately kept parsimonious, and the results should be interpreted with caution.

## 4. Discussion

In this study, we evaluated the association between RWPT obtained from a 12-lead surface ECG and poor CCC in patients with CTO. We found that RWPT, together with age, was independently associated with impaired CCC. Our findings suggest that RWPT may reflect ischemia-related alterations in ventricular activation and may provide adjunctive, non-invasive information, rather than serving as a definitive diagnostic marker of myocardial perfusion.

The association between prolonged RWPT and the severity of coronary artery disease may be partly explained by the impact of ischemia on ventricular conduction. Myocardial oxygen deprivation can slow conduction within both the Purkinje network and ventricular myocytes, thereby prolonging the time required for electrical activation to travel from the endocardium to the epicardium of the heart. This results in a potential increase in RWPT on the surface ECG [18,19]. The degree of ventricular activation delay may parallel the severity of ischemia; thus, RWPT prolongation could be related to the anatomical complexity and functional significance of coronary stenosis [20]. Furthermore, microcirculatory impairment may exacerbate ischemia and further slow intraventricular conduction [21]. Compared to other echocardiographic or ECG markers, its advantage lies in its potential sensitivity to early phase ischemic changes [22]. Whereas the QRS duration represents the total ventricular depolarization time, the RWPT specifically reflects the initial component of ventricular activation, which may be more susceptible to ischemic delay [23].

Previous studies have reported that, in patients with CTO without prior myocardial infarction, good CCC classified as Rentrop grade 3 markedly reduces resting segmental wall motion abnormalities and perfusion defects compared with Rentrop grades 1–2 [24]. Wustmann et al. also demonstrated that, in approximately one-quarter of healthy individuals, CCC channels can mitigate ischemia during brief episodes of coronary occlusion [25]. These findings indicate that a functionally adequate CCC network plays a central role in preserving myocardial perfusion and limiting ischemic burden in patients with CTO. Because CCCs are the sole source of distal perfusion in a totally occluded artery, their adequacy is considered a key physiological determinant of the clinical course of CTO. However, it should be acknowledged that coronary collateral circulation in this study was assessed solely using the Rentrop angiographic classification. While Rentrop grading provides a practical and widely accepted anatomical assessment of collateral vessels, it does not necessarily reflect functional collateral flow or true myocardial perfusion. Accordingly, misclassification of CCC adequacy cannot be excluded, particularly in patients with visually apparent but functionally insufficient collaterals. More precise functional assessments, such as collateral flow index measurements, myocardial perfusion imaging, or physiological indices, were not available in the present study. The absence of these modalities represents an important limitation and may have influenced the observed associations.

ECG is a simple, widely used, non-invasive diagnostic tool in clinical practice. Several studies have demonstrated associations between various ECG parameters, such as fQRS [26] and Tp–e interval [27], and CCC status in patients with CTO. Although RWPT has been shown to correlate significantly with the severity of coronary artery disease, data specifically addressing its relationship with CCC in CTO patients remain limited [28]. The pathophysiology of RWPT is likely multifactorial. In addition to increased dispersion caused by prolonged action potential duration and alterations in calcium and potassium ion conductance, reduced gap junction density, increased interstitial fibrosis, and enlargement of cardiac myocytes may all contribute to RWPT prolongation [29].

We hypothesized that ischemia-related ventricular activation delay in Purkinje fibers and ventricular myocytes would be more pronounced in patients with CTO and poor CCC, leading to greater prolongation of RWPT. Our results demonstrated that RWPT was significantly longer in patients with poor CCC and remained independently associated with impaired CCC. Although LVEF and LV dimensions differed between groups, RWPT remained independently associated with poor CCC after adjustment, suggesting that the observed relationship was not solely driven by overt left ventricular structural or systolic differences.

Similarly, although QRS duration appeared to be significant in the univariate analysis, it did not remain an independent variable in the multivariate analysis. This finding suggests that RWPT may capture earlier and more subtle phases of ventricular depolarization delay associated with ischemia.

In our study, the finding that RWPT remained associated with poor CCC, independent of age, is noteworthy. Although age is well known to influence the development of CCC, the persistence of RWPT as a significant variable in the model suggests that this parameter may provide incremental information regarding ischemia-related ventricular activation delay.

From a clinical perspective, the fact that RWPT can be easily obtained from a routine ECG without additional cost suggests that it may serve as a useful adjunctive parameter in the initial evaluation of patients with CTO. From a clinical perspective, the fact that RWPT can be easily obtained from a routine ECG without additional cost suggests that it may serve as a useful adjunctive parameter in the initial evaluation of patients with CTO. Although RWPT demonstrated a moderate discriminative ability in ROC analysis, this performance should be interpreted cautiously, and RWPT should be regarded as a hypothesis-generating rather than a standalone indicator of CCC adequacy, given the cross-sectional design, potential confounders, and limited sample size.

It should also be noted that RWPT may be influenced by multiple physiological and pathological factors, including heart rate, ventricular hypertrophy, conduction abnormalities, and metabolic or ischemic conditions, which may contribute to variability across different clinical settings In addition, the relatively small sample size and limited number of outcome events may increase the risk of model overfitting and coefficient instability, and therefore the multivariable analysis should be regarded as exploratory rather than confirmatory. Larger prospective studies are needed to confirm these findings.

## 5. Limitations

This study had several limitations. First, it had a single-center design, which may have introduced potential bias in patient selection and data recording processes. Second, CCC was assessed using the Rentrop angiographic classification, which reflects anatomical visualization rather than functional collateral flow, and therefore misclassification of collateral adequacy cannot be excluded. Third, the number of patients included was relatively limited; validation studies conducted on larger populations would enhance the generalizability of the findings.

Fourth, the assessment of CCC was based on angiographic appearance, which may not fully reflect CCC function. Advanced imaging modalities, such as myocardial perfusion imaging, FFR-CT, or invasive hemodynamic assessment, were not used.

Fifth, RWPT was measured using surface ECG recordings, and although standard techniques were applied to minimize variability, a minimal degree of observer-dependent variation in ECG interpretation remains possible.

## 6. Conclusions

This study demonstrated that RWPT obtained from a 12-lead surface ECG was significantly and independently associated with poor CCC in patients with CTO. Along with age, RWPT remained one of the variables that were independently associated with poor collateralization in multivariate analysis.

These findings suggest that RWPT, as a simple, easily obtainable, and non-invasive parameter, may provide adjunctive information regarding CCC status and should be interpreted in the context of clinical findings and other diagnostic modalities.

## Figures and Tables

**Table 1:** Baseline demographic, clinical, laboratory, and electrocardiographic characteristics of the study population stratified by CCC flow status.

Variable	Good CCC (*n* = 52)	Poor CCC (*n* = 40)	*p* Value
Demographic Data			
Male sex, *n* (%)	47 (90.4)	35 (87.5)	0.455
Age (years)	60.2 ± 7.9	64.1 ± 10.1	0.043
Hypertension, *n* (%)	35 (67.3)	31 (77.5)	0.200
Hyperlipidemia, *n* (%)	23 (44.2)	17 (42.5)	0.519
Diabetes mellitus, *n* (%)	18 (34.6)	14 (35.0)	0.571
Body mass index (kg/m^2^)	29.5 ± 4.4	29.1 ± 4.4	0.487
Smoking, *n* (%)	26 (50.0)	22 (55.0)	0.396
Previous stent, *n* (%)	29 (55.8)	22 (55.0)	0.554
Angiographic Data			
LAD CTO, *n* (%)	8 (15.4)	9 (22.5)	0.273
CX CTO, *n* (%)	10 (19.2)	9 (22.5)	0.389
RCA CTO, *n* (%)	34 (65.4)	23 (57.5)	0.289
Number of CTO lesions	1.07 ± 0.23	1.13 ± 0.25	0.082
Medication Use			
Aspirin, *n* (%)	35 (67.3)	24 (60.0)	0.306
ACE inhibitors, *n* (%)	19 (36.5)	15 (37.5)	0.548
ARB, *n* (%)	13 (25.0)	10 (25.0)	0.598
Beta-blockers, *n* (%)	20 (38.5)	17 (42.5)	0.429
Calcium channel blockers, *n* (%)	9 (17.3)	7 (17.5)	0.597
Statins, *n* (%)	12 (23.1)	11 (27.5)	0.402
Oral antidiabetics, *n* (%)	15 (28.8)	12 (30.0)	0.542
Insulin, *n* (%)	4 (7.7)	2 (5.0)	0.470
Laboratory Measurements			
Glucose (mg/dL)	122.0 ± 38.9	126.3 ± 57.6	0.666
Serum creatinine (mg/dL)	0.97 ± 0.21	0.99 ± 0.25	0.833
Total cholesterol (mg/dL)	204.1 ± 55.4	203.5 ± 38.9	0.953
LDL cholesterol (mg/dL)	135.2 ± 44.8	133.4 ± 37.6	0.831
HDL cholesterol (mg/dL)	42.6 ± 7.2	43.5 ± 8.9	0.686
Triglycerides (mg/dL)	182.1 ± 81.2	136.8 ± 54.2	0.003
WBC (×10^3^/µL)	8.7 ± 2.3	9.3 ± 2.7	0.263
Neutrophils (×10^3^/µL)	5.7 ± 1.8	6.4 ± 2.6	0.136
Hemoglobin (g/dL)	14.4 ± 1.9	14.1 ± 1.9	0.148
Electrocardiographic Data			
Interatrial block, *n* (%)	8 (16.3)	7 (17.5)	0.552
Pathologic Q waves, *n* (%)	15 (28.8)	18 (45.0)	0.084
Fragmented QRS, *n* (%)	16 (30.8)	16 (40.0)	0.241
QRS duration (ms)	93.1 ± 9.8	98.3 ± 11.3	0.021
P-wave dispersion (ms)	45.9 ± 4.1	46.9 ± 4.6	0.290
QTc interval (ms)	388.6 ± 34.3	397.7 ± 43.9	0.606
QT interval (ms)	415.8 ± 25.1	418.9 ± 32.4	0.268
R-wave peak time (ms)	34.1 ± 7.7	40.5 ± 12.5	0.002
Tp interval (ms)	316.7 ± 107.4	303.1 ± 116.8	0.570
Tp–e duration (ms)	103.5 ± 10.2	104.5 ± 10.1	0.639
Heart rate (bpm)	70.8 ± 12.9	72.9 ± 15.6	0.470
PTF-V1	51.8 ± 21.6	55.7 ± 23.6	0.425
LVEF (%)	52.5 ± 7.9	47.0 ± 10.2	0.006
LVEDD (mm)	49.3 ± 3.7	51.2 ± 5.8	0.018
LVESD (mm)	33.3 ± 3.8	36.5 ± 7.5	0.043
IVS thickness (cm)	1.19 ± 0.14	1.22 ± 0.21	0.429
Posterior wall thickness (cm)	1.08 ± 0.10	1.11 ± 0.15	0.455

Values are presented as mean ± standard deviation or number (percentage). CTO: chronic total occlusion; LDL: low-density lipoprotein; HDL: high-density lipoprotein; WBC: white blood cell count; Tp–e: Tpeak–Tend interval; PTF-V1: P-wave terminal force in V1.

**Table 2:** Univariate and multivariate logistic regression analyses identifying predictors of poor CCC.

Variable	Univariate OR	95% CI	*p* Value	Multivariate OR	95% CI	*p* Value
Age (years)	1.047	1.001–1.098	0.037	1.058	1.005–1.114	0.033
* Triglycerides (mg/dL)	0.989	0.982–0.997	0.007			
* QRS duration (ms)	1.071	1.022–1.123	0.004			
R-wave peak time (ms)	1.049	1.006–1.093	0.025	1.069	1.013–1.128	0.015

* Not statistically significant in the multivariate analysis (dropped in backward elimination). OR: odds ratio; CI: confidence interval.

**Table 3:** Parsimonious multivariable logistic regression model for poor coronary collateral circulation.

Variable	Odds Ratio (OR)	95% Confidence Interval	*p* Value
Age (years)	1.06	1.00–1.11	0.039
Triglyceride (mg/dL)	0.99	0.98–1.00	0.073
cRPT (Fridericia, ms)	1.06	1.01–1.11	0.016

Model statistics: Likelihood ratio *p* < 0.001; Pseudo R^2^ = 0.15. The model was constructed using a parsimonious approach including clinically relevant variables to reduce the risk of overfitting. Results should be interpreted as exploratory.

## Data Availability

The data presented in this study are available upon reasonable request from the corresponding author.

## Supplementary Materials

The following supporting information can be downloaded at https://www.mdpi.com/article/10.3390/jcm15020450/s1, File S1: Definitions and Measurement of Electrocardiographic Parameters.
