# Supplementary material for: R-Wave Peak Time and Impaired Coronary Collateral Circulation in Chronic Total Occlusion
Source: J Clin Med. 2026 Jan 7;15(2):450. doi: 10.3390/jcm15020450 (PMC12841970; doi:10.3390/jcm15020450)
Supplement: Supplementary file 1 [file jcm-15-00450-s001.zip › jcm-4051341-supplementary.pdf]

QRS duration was defined as the time interval between the onset of the q or R wave and the end of the S wave (J point) and was measured manually using digital calipers on the 12-lead surface electrocardiography (ECG) tracings. The longest QRS duration among leads V1 to V6 was recorded.

Fragmented QRS was examined by analyzing the QRS complex in various leads to identify the presence of fragmented QRS patterns. Fragmented QRS is characterized by the existence of additional deflections or notches within the QRS complex, which indicate abnormal depolarization patterns. The presence and characteristics of fragmented QRS patterns in the corresponding leads were documented.

The presence of pathological Q waves on the ECG was identified. The duration and depth of the Q wave were measured in the leads where it appeared most prominently. Pathologic Q waves were defined as having a duration greater than 40 milliseconds and a depth exceeding 25% of the subsequent R wave or S wave.

Inter-atrial Block was assessed by examining the morphology of the P wave in various leads. The investigation focused on identifying prolonged or altered P wave duration, morphology, or axis deviation. Inter-atrial block was diagnosed using specific criteria, including a P wave duration exceeding 120 milliseconds, with or without a biphasic negative P wave in leads II, III, and aVF.

R-wave peak time was assessed by measuring the duration from the onset of the QRS complex to the peak of the R wave. The initial deflection point of the QRS complex was identified, and the peak of the R wave was located. The time interval between these two points was quantified in milliseconds.

P (Maximum) and P (Minimum) were determined by identifying the maximum and minimum durations of the P wave. The onset and offset points of the P wave were located in a

lead where it was most prominent. The time intervals between these points were measured in milliseconds. P Dispersion was calculated by subtracting the minimum duration of the P wave from the maximum duration. This calculation provided a measure of P wave dispersion across different leads, and the result was reported in milliseconds.

P-wave terminal force in V1 was determined locally by calculating the absolute value of the depth of the P-wave's downward deflection in lead V1 on an ECG and subsequently multiplying it by the duration of the deflection.

QT Interval was measured by identifying the onset and offset points of the QT interval. The duration of the QT interval was measured in milliseconds, spanning from the beginning of the Q wave to the end of the T wave. QTc Interval was measured as the duration from the onset of the Q wave to the end of the T wave. To account for heart rate, Bazett's formula ($QTc = QT / \sqrt{RR}$) was employed, using the RR interval. The resulting QTc interval was calculated and reported in milliseconds.

TP Interval was measured from the end of the T wave to the onset of the subsequent P wave. This interval represents the period from the end of ventricular repolarization and the beginning of atrial depolarization. TP-e duration was measured as the duration from the peak of the T wave to the end of the T wave in leads V2 or V5, where the T wave exhibited the most prominent features.
